# Therapeutic effects of 3% diquafosol ophthalmic solution in patients with short tear film break-up time-type dry eye disease

**DOI:** 10.1186/s12886-018-0910-3

**Published:** 2018-09-05

**Authors:** Yongseok Mun, Ji-Won Kwon, Joo Youn Oh

**Affiliations:** 1 Department of Ophthalmology, Seoul National University Hospital, 101, Daehak-ro, Jongno-gu, Seoul, 03080 South Korea; 2 Department of ophthalmology, Hanyang University College of Medicine Myongji Hospital, 14 Bungil 55, Hwasu-Ro, Deokyang-Gu, Goyang-si, Gyeonggi-do 14075 South Korea; 3 Laboratory of Ocular Regenerative Medicine and Immunology, Biomedical Research Institute, Seoul National University Hospital, 101 Daehak-ro, Jongno-gu, Seoul, 03080 South Korea

**Keywords:** Diquafosol tetrasodium 3%, Dry eye disease, Short tear film break-up time-type dry eye

## Abstract

**Background:**

To investigate therapeutic effects of topical diquafosol tetrasodium 3% ophthalmic solution in patients with short tear film break-up time (TFBUT)-type dry eye (DE).

**Methods:**

The prospective study was performed in 70 eyes of 70 patients with short TFBUT-type DE. Diagnosis of short TFBUT-type DE was made based on the presence of DE symptoms, TFBUT value ≤5 s, corneoconjunctival staining score ≤ 2 (on a scale of 0 to 4), and Schirmer I value > 5 mm. Patients with systemic immunologic disorders or ocular graft-versus-host disease were excluded. Before and after instillation of 3% diquafosol ophthalmic solution six times per day for 4 weeks, subjective DE symptoms, TFBUT, corneoconjunctival staining score, and Schirmer I value were examined and compared. Also, demographic factors were compared between patients who showed improvement in each DE parameter by treatment and those who did not.

**Results:**

Four-week treatment with 3% diquafosol ophthalmic solution significantly improved DE symptoms (*p* < 0.0001), increased TFBUT (*p* < 0.0001), and reduced corneoconjunctival staining scores (*p* < 0.0001). Schirmer I values were not changed by treatment. The age of patients who showed improvement in subjective DE symptoms after treatment was significantly lower than that of patients who did not (53.4 ± 27.5 vs. 63.3 ± 13.9 years, *p* = 0.012). Ocular side effects developed in 3 patients (4.3%), including conjunctival chemosis (*n* = 1) and persistent stinging sensation (*n* = 2).

**Conclusions:**

Diquafosol tetrasodium 3% ophthalmic solution is effective in improving subjective symptoms and tear film stability in short TFBUT-type DE patients.

**Trial registration:**

The study was retrospectively registered on Clinical Research Information Service (CRiS), Republic of Korea.

Trial registration number: KCT0003134.

Date of registration: 2018-08-15.

## Background

Dry eye disease (DED) is a multifactorial disease of the ocular surface, which is associated with various conditions such as tear film instability and hyperosmolarity, ocular surface inflammation and damage, or neurosensory abnormalities [1]. For etiopathogenic classification of DED, the TFOS DEWS II Definition and Classification Subcommittee reinforced the two major etiological categories of DED: aqueous deficient and evaporative [1]. Aqueous deficient dry eye (ADDE) is due to a failure of lacrimal tear production, and evaporative dry eye (EDE) is associated with increased evaporation of the tear film in the presence of normal lacrimal secretion. Importantly, ADDE and EDE are not mutually exclusive and commonly overlapping, [1] both leading to tear film instability which can be measured by the tear break-up time (TFBUT). Recently, a new concept of short tear film break-up time (TFBUT)-type dry eye (DE) has emerged as a potentially new type of DED [2, 3].

The short TFBUT-type DE is characterized by a short TFBUT and DE symptoms without ocular surface damage and tear deficiency [2, 3]. This type of DE has been shown to be associated with visual display terminal (VDT) work and contact lens (CL) wear [4–7], and therefore, the prevalence of short TFBUT-type DE is rising with the widespread use of VDT and CL [8, 9]. For the treatment, a few studies in a small number of patients showed the efficacy of 3% diquafosol ophthalmic solution in improving subjective symptoms and TFBUT in patients with short TFBUT-type DE [10–12].

Diquafosol is an agonist for P2Y_2_ purinergic receptor which exists in conjunctival epithelial cells, goblet cells, and epithelium of meibomian glands [13–16]. By activating P2Y_2_ receptors on the ocular surface, diquafosol stimulates both fluid secretion from conjunctival epithelial cells and mucin secretion from goblet cells, thereby stabilizing the tear film and hydrating the ocular surface, independent of tear secretion from lacrimal glands [13–16]. Hence, it is possible that diquafosol ophthalmic solution has therapeutic potential in short TFBUT-type DE patients. In this study, we investigated the therapeutic effects of topical application of diquafosol tetrasodium 3% ophthalmic solution in patients with short TFBUT-type DE.

## Methods

### Patients

The study was approved by the Institutional Review Board of Seoul National University Hospital (IRB No. 1402–058-557) on February 25, 2014 and performed in accordance with the tenets of the Declaration of Helsinki. The patients with short TFBUT-type DE were prospectively enrolled with informed consent in Seoul National University Hospital from March 14, 2014 to December 19, 2014 and followed-up for 4 weeks. The diagnosis of short TFBUT-type DE was made based upon the presence of DE symptoms, TFBUT value ≤5 s, corneoconjunctival staining score ≤ 2 (on a scale of 0 to 4), and Schirmer I test value > 5 mm/5 min. Patients with systemic immune-mediated diseases such as secondary Sjögren’s syndrome or graft-versus-host disease were excluded. Also, patients who were on topical medication(s) for treatment of ocular diseases other than DED such as glaucoma or allergic conjunctivitis were excluded.

### Treatment and examination

The subjective DE symptoms, TFBUT, corneoconjunctival staining scores, and Schirmer I values were examined before and after 4 weeks of instillation of diquafosol tetrasodium 3% ophthalmic solution (Diquas®, Santen Pharmaceutical Co., Ltd., Osaka, Japan) six times per day. Other topical medications than Diquas® were discontinued during the study period. Demographic factors including age and gender were compared between patients who showed improvement in each DE parameter after treatment and those who did not show any change. Systemic or ocular side effects were recorded.

The criteria for subjective DE symptom scoring are as follows: 0 (no symptom), 1 (mild symptoms of ocular dryness, occasional and not interfering with daily life), 2 (moderate symptoms, occasional or chronic, but not interfering with daily life), 3 (severe frequent or constant, but not interfering with daily life), and 4 (severe, constant and disabling). The corneoconjunctival staining score was assessed by fluorescein staining as follows: 0 (no staining), 1 (mild conjunctival and corneal staining), 2 (moderate conjunctival and corneal staining), 3 (moderate to severe conjunctival staining and marked central corneal staining), and 4 (severe conjunctival staining and severe, diffuse corneal punctate erosions).

TFBUT measurements were performed as previously recommended [2]. In brief, after instillation of a small quantity of 1% fluorescein dye into the inferior conjunctival sac, patients were instructed to blink several times to ensure mixing of the dye with the tear. Under a cobalt blue filter and slit lamp biomicroscope, the interval between the last complete blink and appearance of the first corneal black spot in the stained tear film was measured [2]. The measurements were made three times for each eye by the same doctor, and the mean value of the three measurements was calculated.

The Schirmer I test was done by putting the Schirmer paper strip at the junction of the middle and lateral thirds of the lower eyelid of patients without anesthesia. Patients were then instructed to close their eyes for 5 min, and the wetting length of the strip was recorded in mm.

The primary outcome was the mean change from baseline in TFBUT at 4 weeks after treatment and the secondary outcome measures were the changes in the subjective DE symptom scores and Schirmer I test results.

### Statistical analysis

The power analysis was performed to justify the number of patients using G*Power 3.1.9.2. A sample size of 80 patients was determined to provide the study with 80% statistical power to detect 30% mean difference in TFBUT between control and treatment groups under the assumption that data would be missing in 15% of patients.

The GraphPad Prism® (GraphPad Software, Inc., La Jolla, CA) was used for statistical analysis. The change between pre and post-treatment values in each patient was assessed for significant difference by two-tailed paired *t* test. The comparisons of demographic factors between patients who showed improvement in each DE parameter after treatment and those who did not were made by two-tailed *t* test. Statistical significance was defined as *p* value < 0.05.

## Results

Initially, 80 eyes of 80 patients with short TFBUT-type DE were enrolled. In case of bilateral involvement, the eye with shorter TFBUT at baseline was included for analysis. Three eyes were withdrawn from the study because of ocular side effects and 7 eyes excluded because of follow-up loss. Therefore, a total of 70 eyes completed the study, from which data were analyzed.

Demographic data and baseline ocular characteristics were shown in Table 1. The subjects included 9 men and 61 women, and the mean age was 55.8 ± 14.1 years (range 22 to 83 years). At the time of enrollment, 27 patients (38.6%) were using artificial tears containing hyaluronic acid on irregular basis without symptomatic relief. The subjective DE symptom score was 2.84 ± 0.65 at baseline, and TFBUT was 2.72 ± 0.94 s. The corneoconjunctival staining score was 1.17 ± 0.61, and Schirmer I value was 9.34 ± 2.60 mm. Patients were instructed to discontinue any eye drops and start using 3% diquafosol ophthalmic solution alone.

**Table 1:** Patient demographics and baseline ocular characteristics (*n* = 70)

Demographics	
Gender (male: female)	9: 61
Age (years in mean ± SD, range)	55.8 ± 14.1 (22–83)
Diabetes mellitus	4 (5.7%)
Use of topical medication(s)	
Artificial tears (hyaluronic acid-based)	27 (38.6%)
Ocular characteristics	
Dry eye symptom score (range)	2.84 ± 0.65 (1–4)
Tear film break-up time (sec, range)	2.72 ± 0.94 (1–5)
Ocular staining score (range)	1.17 ± 0.61 (0–2)
Schirmer I test (mm, range)	9.34 ± 2.60 (6–18)

**Fig. 1:**
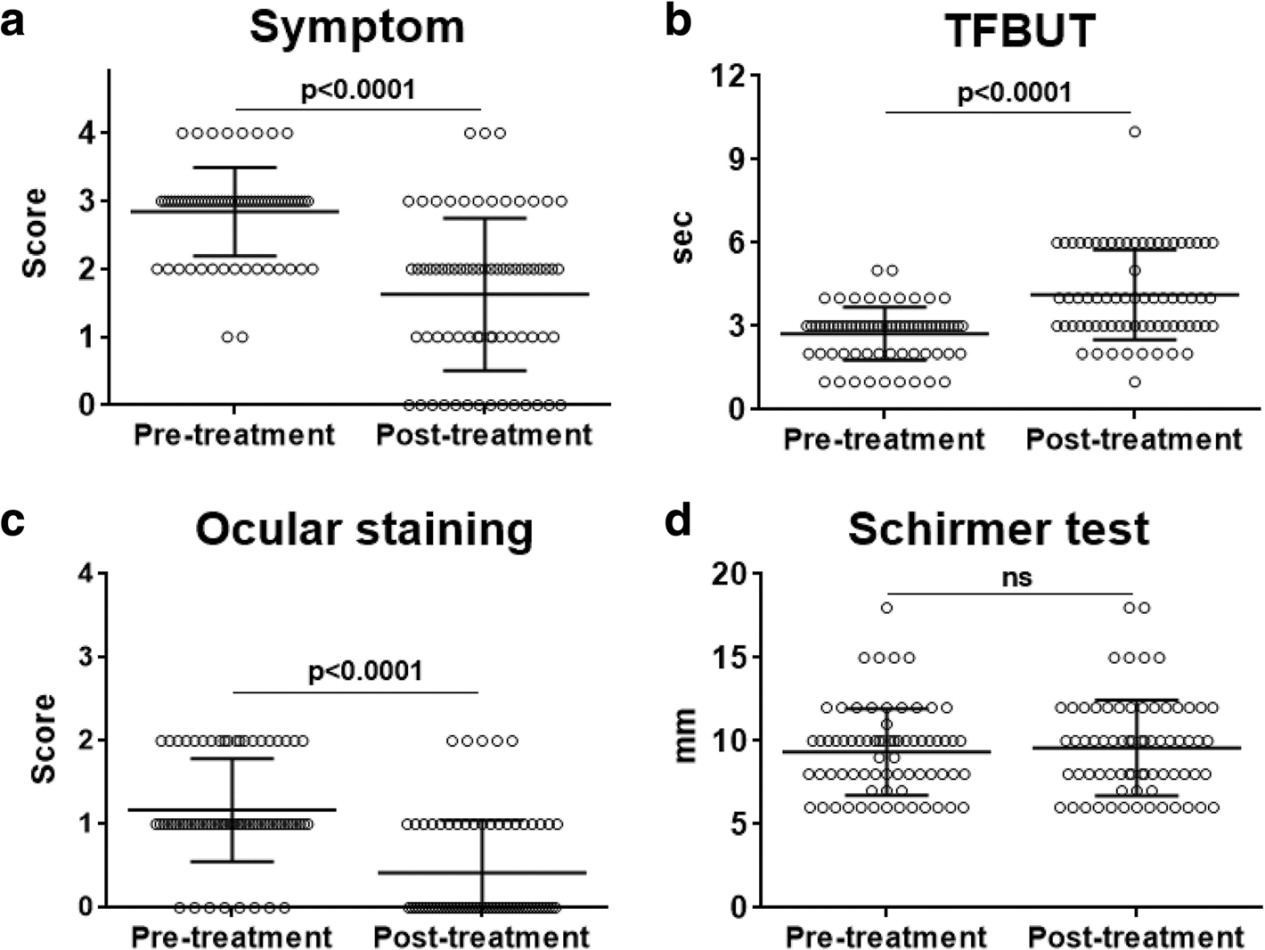
Comparison of changes in dry eye parameters before and after 3% diquafosol ophthalmic solution administration. Changes in subjective dry eye symptom scores (**a**), tear film break-up time (TFBUT) (**b**), corneoconjunctival staining scores (**c**), and Schirmer I test values (**d**) before (pre-treatment) and after 4 weeks of 3% diquafosol ophthalmic solution administration (post-treatment)

After 4 weeks of treatment with 3% diquafosol ophthalmic solution, the subjective DE symptom improved in 55 eyes (78.6%) compared to before treatment, and TFBUT increased in 46 eyes (65.7%) (Table 2). The corneoconjunctival staining score was reduced in 44 eyes (62.8%) by treatment (Table 2). Quantitative analysis showed that the diquafosol treatment significantly reduced DE symptom scores in patients from 2.84 ± 0.65 to 1.63 ± 1.19 (*p* < 0.0001) and increased TFBUT from 2.72 ± 0.94 s to 4.13 ± 1.62 s (*p* < 0.0001) (Fig. 1). Also, the corneoconjunctival staining scores decreased from 1.17 ± 0.61 to 0.42 ± 0.63 after treatment (*p* < 0.0001) (Fig. 1). However, the Schirmer I values were not changed by treatment (9.34 ± 2.60 mm before treatment vs. 9.59 ± 2.88 mm after treatment, *p* = 0.6013).

**Table 2:** The percentage of patients who showed changes in dry eye symptoms and ocular signs after 4 weeks of topical 3% diquafosol ophthalmic solution administration

Changes after treatment	No. patients (%)		
	Improved	No change	Worsened
Subjective dry eye symptom	55 (78.6)	15 (21.4)	0 (0)
Tear film break-up time^a^	46 (65.7)	22 (31.4)	2 (2.9)
Ocular staining score	44 (62.8)	23 (32.9)	3 (4.3)

^a^An increase in tear film break-up time after treatment was defined as “Improved, and a decrease as “Worsened”. The same values of tear film break-up time before and after treatment were defined as “No change”

Additionally, demographic factors were compared between patients who showed improvement in each DE parameter and those who did not show any improvement with 3% diquafosol sodium. Of note was the finding that the age of patients whose DE symptoms improved after treatment was significantly lower than that of patients whose symptoms did not change (53.4 ± 27.5 vs. 63.3 ± 13.9 years, *p* = 0.012) (Table 3). No significant changes were found in the age between patients who had improvement in TFBUT or ocular staining score and those who did not (Table 3).

**Table 3:** The age of patients who showed improvement in dry eye symptoms and ocular signs after 4 weeks of topical 3% diquafosol ophthalmic solution administration

Changes after treatment	Age of patients (mean ± SD)		*p* value
	Improved	Not improved	
Subjective dry eye symptom	53.4 ± 27.5	63.3 ± 13.9	0.012
Tear film break-up time^a^	55.5 ± 13.8	56.5 ± 14.5	0.782
Ocular staining score	54.6 ± 13.9	57.8 ± 14.1	0.359

^a^An increase in tear film break-up time after treatment was defined as “Improved, and a decrease as “Worsened”. The same values of tear film break-up time before and after treatment were defined as “No change

Ocular side effects developed in 3 patients (4.3%) that included persistent stinging sensation in two patients and conjunctival chemosis in one. Conjunctival chemosis disappeared after cessation of diquafosol and application of fluorometholone eye drops (Flarex®, Alcon Laboratories, Inc. Fort Worth, TX). The stinging sensation completely resolved in all patients after discontinuing 3% diquafosol.

## Discussion

Since 3% diquafosol ophthalmic solution (Diquas®) was launched and approved in South Korea (in 2011) and in Japan (in 2010), it has been widely used in clinic for treatment of DE. As a P2Y_2_ purinergic receptor agonist, diquafosol has novel mechanisms of action including stimulation of fluid and mucin secretion in conjunctival epithelial and goblet cells or promotion of membrane-binding mucin gene expression in corneal epithelial cells [13–16]. Therefore, diquafosol increases fluid secretion independent of lacrimal gland function and makes the ocular surface more hydrophilic, both of which lead to strengthening of the aqueous-mucin layer and stabilization of tear film in healthy subjects and DE patients [13–16].

Topical instillation of 3% diquafosol ophthalmic solution has been reported to be effective for both categories of DED: ADDE and EDE [14–20]. In particular, several studies recently reported that topical diquafosol is effective in alleviating symptoms and increasing TFBUT in patients with short TFBUT-type DE [10–12] which is characterized by a reduced TFBUT without much epithelial damage and lacrimal gland dysfunction. In a study by Shimazaki-Den et al. [10], 39 eyes with DE symptoms and short TFBUT, but without epithelial damage and decreased Schirmer I values (≤ 5 mm), received 3% diquafosol ophthalmic solution 6 times a day. The subjective symptoms as assessed by a VAS (visual analog scale) score and TFBUT significantly improved from baseline at 1 and 3 months after treatment, whereas there was no significant change in Schirmer values. In another study by Jung et al. [11], 30 DED patients who had TFBUT < 5 s and basal tear secretion ≥5 mm on the Schirmer test were treated with 3% diquafosol 6 times a day in combination with 0.1% hyaluronic acid artificial tears. At 1 and 3 months, TFBUT was increased and DE symptoms decreased, although there were no differences in corneoconjunctival staining scores and Schirmer test results. In line with these studies, we here investigated clinical effects of 3% diquafosol in 70 patients with short TFBUT-type DE. Results demonstrate that topical application of 3% diquafosol for 1 month provided improvement of DE symptoms, TFBUT, and corneoconjunctival staining in 78.6%, 65.7%, and 62.8% of short TFBUT-type DE patients, respectively. Compared to before treatment, TFBUT was significantly increased after treatment, and DE symptoms and corneoconjunctival staining scores significantly decreased. Ocular adverse reactions were observed in 4.3% of patients, which is comparable to 6.3% in previous studies [17].

There are several novel findings in our study. One is that the age of patients who had symptomatic relief in response to diquafosol treatment was lower compared to patients who did not. Therefore, it is possible that diquafosol might serve as an effective treatment for short TFBUT-type DE related to environmental stresses such as VDT use or CL wear in young patients with normal lacrimal gland function [4–7]. Another is that the patients in our study had severe symptoms (the mean symptom score 2.84 on a scale of 0 to 4) relative to mild ocular surface damage (the mean staining score 1.17 on a scale of 0 to 4). All of them had normal lacrimal gland secretion (Schirmer I values > 5 mm), and the ocular surface inflammation was not clinically evident in most of patients. This type of DED corresponds to the level 1 and 2 DED according to the Korean Corneal Disease Study Group guidelines for the diagnosis of dry eye disease [21], and represents the most common type of DED in real world [2, 21]. Hence, our study suggests a possibility that 3% diquafosol ophthalmic solution might have a broader implication as a therapy for DED patients. In support for this, a multicenter, prospective, non-interventional observational study involving over 3000 “real-world” DED patients demonstrated that administration of 3% diquafosol ophthalmic solution was effective regardless of the severity of DED based on the ocular staining score, and a total of 76.0% of the enrolled patients responded that their condition had improved after 2 months of treatment [17]. Similarly, 3% diquafosol provided symptomatic relief in 78.6% of patients in our study.

Our study has several limitations. First, the treatment was not compared with a control treatment in parallel. Second, the therapeutic outcome and side effects were examined in the short-term (1 month after treatment). Third, it was performed in a small number of patients (*n* = 70), although our study involves the largest number of short TFBUT-type DE patients where the effects of diquafosol have been tested so far. In the future, a randomized controlled study in a larger number of patients with the longer follow-up period would be necessary to identify the DED type in which diquafosol is the most effective and to further broaden the disease indications for its use.

## Conclusions

In conclusion, diquafosol tetrasodium 3% ophthalmic solution is effective in alleviating symptoms, stabilizing tear film, and decreasing ocular surface damage in patients with short TFBUT-type DE.

## Abbreviations

ADDE: aqueous tear-deficient dry eye
CL: contact lens
DE: dry eye
DED: dry eye disease
EDE: evaporative dry eye
TFBUT: tear break-up time
VAS: visual analog scale
VDT: visual display terminal
